# The Report of the Medical Officer of Health of the County of London

**Published:** 1909-02-20

**Authors:** 


### Public Health and Hygiene.

### THE REPORT OF THE MEDICAL OFFICER OF HEALTH OF THE COUNTY OF LONDON.

The reports of Sir Shirley Murphy are always interesting, and indeed, when the vital conditions of the unique population dealt with by the Medical Officer of the County of London are considered they cannot fail to be so. The present volume reflects the vital circumstances of the population of London not only during the year under review, but, as in previous reports, over a considerable period, the graphic settings permitting a comparison of over sixty years of carefully-prepared statistical returns.

We learn at a glance that the marriage rate continues its somewhat erratic decline; that the birth rate persists in its steady, consistent, and, on the whole, increasing rate of fall; that the general death rate with less consistency, but not less decisively, diminishes at a pace which threatens its extinction.

The analysis of these mortality returns, so that their significance shall be made clear, is a task which can only be appreciated when the magnitude of the data are comprehended. To take only one instance: it might be supposed that the fall in the London death rate is chiefly to be ascribed to changes in the age or sex constitution of London's population. But a comparative table showing for each sex the death rates at several age periods in 1907, as compared with those of the decennium 1891-1900, shows that for each sex at each age period of life, except the period 85 and upwards, the rate of mortality has declined considerably—the difference for all ages being a reduction of over 24 per cent. It is interesting to learn from Sir Shirley Murphy's calculations that this represents in persons at all ages a saving of 22,146 lives, and in life capital of 905,505 years.

At a time when municipal activities are measured almost exclusively by the criterion of "the rates" it is well that this colossal gain should not escape attention. The money actually expended in public health administration, though it is much talked about, forms relatively an insignificant proportion of municipal expenditure, and its results in relation to their importance receive only the most meagre acknowledgment. Nearly a million years of life added to London's population is a wonderful achievement—all the more marvellous that it has come in a period of profound change, when the natural conditions of life are being supplanted and replaced by the complex and intricate artifice demanded in the contemporary process of urbanising society.

Perhaps the most striking indication that is given of the limits imposed upon administrative effort is to be found in Sir Shirley Murphy's interesting comparisons of the vital statistics of selected areas in London. The tables show that it is in the older Central London that high death rates are maintained, although in almost all parts of London a marked decline is observable. Curiously, in the borough which shows the highest death rate there is an exception to the otherwise universal rule of a decline in mortality when the year under review is compared with the quinquennium immediately preceeding. Sir Shirley Murphy institutes a comparison between this borough—Shoreditch—with a corrected death rate in 1907 of 21.5, and Hampstead, which has the enviable distinction of yielding the lowest London death rate—10.2 per 1,000.

"In view of these wide differences it is of interest to compare these two districts from the point of view of the 'life expectation' of their populations—in other words to translate their mortality rates into figures showing 'life expectation' at certain ages, and thus to show, by a method which admits of more accurate comparison, the marked differences in the viability of the two populations concerned. The widely different character of the two populations under consideration as regards social condition may to some extent be gauged by reference to the Census Report of the year 1901. Thus, in Hampstead the percentage of domestic indoor servants to families or separate occupiers was as high as 81.4, while in Shoreditch the figure was only 5.7. Again, the proportion of the population living more than two in a room in tenements of less than five rooms was in Hampstead 6.37 per cent., while in Shoreditch the proportion was 29.95 per cent."

As a result of the comparison, Sir Shirley Murphy shows that at each age period, both for males and females, the "expectation of life" is many years greater for persons living in Hampstead than it is in Shoreditch. At birth the "expectation of life" in Shoreditch is nearly 16 years less than in Hampstead.

"After every allowance has been made for the undoubted advantages which Hampstead, compared with Shoreditch, enjoys by reason of its topographical position, it may be fairly claimed that the figures relating to Shoreditch serve to show how much yet remains to be done for the improvement of the physical well-being of a large section of the community, notwithstanding the material advances which have been made in the direction of the improvement of public health in recent years."

Perhaps no more telling example of the extent to which disease is conditioned by social circumstances could be found than in an incidental result of the comparison, which shows that the septennial mean death rate from phthisis in Hampstead is only 0.78 per 1,000 living, while the corresponding death rate for Shoreditch is 2.16 per 1,000.

So significant a difference in fatal prevalence of a definitely specific disease is dependant upon no single factor: it is an expression of the influence upon pulmonary tubercular infection of all that is embraced in the contemporary attainments of personal and civic hygiene as exemplified in a suburb such as Hampstead.

Hampstead is an example of what can be done by reducing to practice the principles with which the teaching of medicine has for long been identified; Shoreditch presents the problem which the advocates of social and hygienic reform have to solve. Knowing what to do, how is it to be done? Sir Shirley Murphy's figures show what the result would be if it were done.

